# Gene Regulation and Quality Control in Murine Polyomavirus Infection

**DOI:** 10.3390/v8100284

**Published:** 2016-10-17

**Authors:** Gordon G. Carmichael

**Affiliations:** Department of Genetics and Genome Sciences, UCONN Health, Farmington, CT 06030, USA; carmichael@uchc.edu; Tel.: +1-860-679-2259

**Keywords:** quality control, transcription, RNA decay, RNA editing, nuclear retention

## Abstract

Murine polyomavirus (MPyV) infects mouse cells and is highly oncogenic in immunocompromised hosts and in other rodents. Its genome is a small, circular DNA molecule of just over 5000 base pairs and it encodes only seven polypeptides. While seemingly simply organized, this virus has adopted an unusual genome structure and some unusual uses of cellular quality control pathways that, together, allow an amazingly complex and varied pattern of gene regulation. In this review we discuss how MPyV leverages these various pathways to control its life cycle.

## 1. The Virus

**Figure 1:**
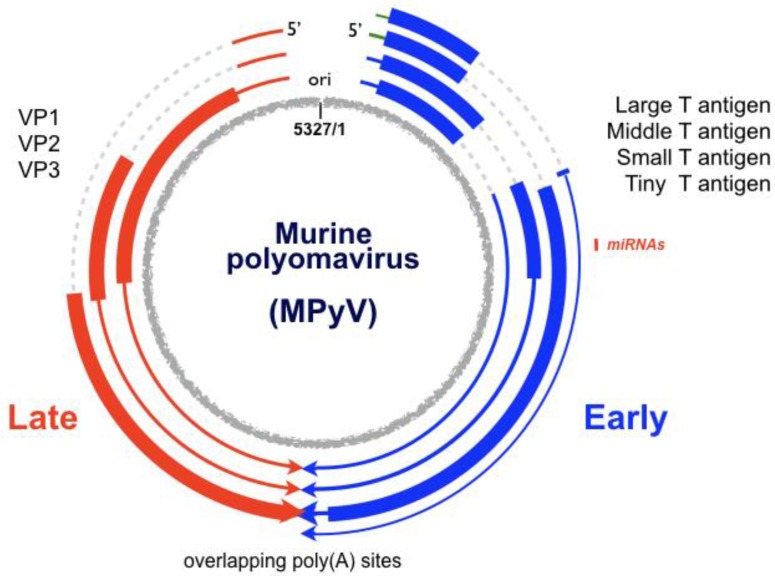
The murine polyomavirus (MPyV) genome. The genome shown is of strain NG59RA, which is 5327 base pairs in length. Early genes are in blue and late genes are in red. Transcripts are lines, with thicker regions denoting open reading frames and dotted lines introns. The replication origin and transcriptional control region is shown at the top of the genome. Late-strand transcripts can give rise to two miRNAs (small red line) that map to the early region and can influence viral and host gene expression.

Murine polyomavirus (MPyV) is highly oncogenic in rodents and has a small circular double-stranded DNA (dsDNA) genome of about 5300 base pairs. The genome is divided into “early” and “late” regions, which are expressed and regulated differently as infection proceeds (Figure 1) [1,2,3,4]. The early and late transcription units extend in opposite directions around the circular genome from start sites near the bidirectional origin of DNA replication [2,5]. Primary RNA products from the early transcription unit are alternatively spliced to yield four early mRNAs which encode the large T antigen (100 kDa), the middle T antigen (56 kDa), the small T antigen (22 kDa) and the tiny T antigen (10 kDa) [6]. Large T binds to sequences in or near the DNA replication origin region [7,8,9,10] and is involved in the initiation of DNA replication, indirectly in the autoregulation of early-strand RNA levels [11,12,13], and indirectly in the activation of high levels of expression from the late promoter [13,14]. The other early proteins are dispensable for lytic infection, but are important for cell transformation and tumorigenesis [15]. Late primary transcripts accumulate after the onset of DNA replication and are also spliced in alternative ways to give mRNAs which code for the three virion structural proteins VP1, VP2 and VP3. 

**Figure 2:**
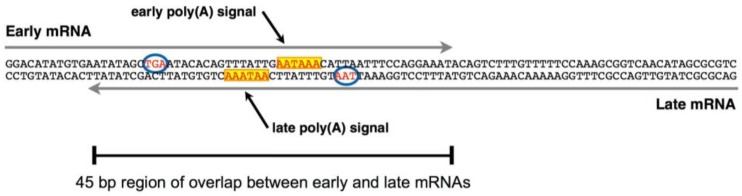
A crowded arrangement at the ends of the early and late genes. The sequence shown is of the 3′-region of the early and late transcription units, with the early coding strand on top and the stop codons for large T antigen and the virion structural protein VP1 circled. Note the overlap of the polyadenylation signals, including the canonical AATAAA elements (yellow box). Cleavage and polyadenylation occur downstream of these elements, leading to early and late mRNAs that have the potential to overlap for at least 45 base pairs (bp) at their 3′-ends. Transcript overlap is essential for the viral life cycle.

While seemingly simply organized, MPyV has adopted an unusual genome structure that provides a platform for the participation of a number of cellular gene regulatory and quality control mechanisms. First, the intergenic region is complex and crowded and serves multiple functions during infection. Consisting of only several hundred nucleotides, this region contains the origin of bidirectional DNA replication, the early promoter and the late promoter. Each of these is impacted by distinct molecular machinery, competing for overlapping sequence elements. Activation of the replication origin requires the recruitment of the cellular DNA replication machinery by large T antigen, which recognizes a number of sites in this region. The early promoter is a typical RNA polymerase II promoter, including a TATA box to specify early transcription start sites and an upstream enhancer region. The late promoter is TATA-less and specifies transcripts with a multitude of 5’-ends spanning more than 100 nucleotides. Second, the distal ends of the early and late regions are tightly connected (Figure 2) and, as we will see below, this organization plays a major role in the regulation of the viral life cycle. The ends of the coding regions for large T antigen and VP1 are very close to one another, separated from each other by only 23 base pairs. Also, the polyadenylation signals for early-strand and late-strand primary transcripts actually overlap one another. This leads to overlapping 3′-ends of early and late mRNAs and pre-mRNAs, with the amount of overlap being 45 base pairs or greater. As we shall discuss below, transcript overlap appears to be essential for the viral life cycle, since viruses that are constructed to eliminate this overlap fail to enter the late phase of infection [16]. Third, the splicing signals for late mRNAs are arranged in a manner rarely seen in eukaryotic transcripts. In most pre-mRNA molecules, the first splice site encountered is a donor, 5′-splice site. This allows the splicing of the first exon to the second exon. In MPyV late transcripts, the cap-proximal splice site is actually an acceptor, 3′-splice site. This almost unique arrangement turns out to be critical for the viral life cycle. 

## 2. The Viral Early–Late Switch

**Figure 3:**
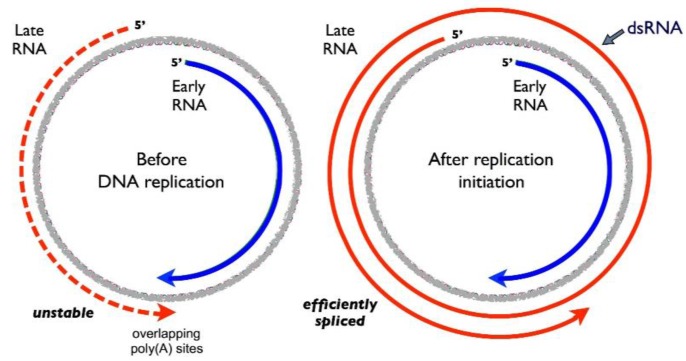
The early–late switch is associated with poly(A) signal readthrough and double-stranded RNA (dsRNA) formation. See text for details of the regulation. At early times and before viral DNA replication (**left**), transcription occurs from both the early and late promoters. Early-strand RNAs are spliced to produce mRNAs for the early proteins. Late-strand transcripts are efficiently terminated and polyadenylated, but are unstable and produce only small amounts of late mRNAs and proteins. After the onset of DNA replication (**right**), transcription termination and polyadenylation become less efficient, allowing multigenomic transcripts to be produced. Giant transcripts are efficiently spliced to generate stable late mRNAs, but sequences antisense to early-strand transcripts can downregulate early genes.

Temporal regulation of MPyV gene expression during lytic infection of permissive mouse cells proceeds in a well-defined and tightly regulated manner [1,17,18]. Immediately after infection, RNA from the early transcription unit begins to accumulate; however, RNA from the late transcription unit fails to accumulate to a significant level. At 12 h after infection, the early–late RNA ratio is about 4 to 1 [1,18,19,20] and in the presence of DNA replication inhibitors, the ratio is 10 to 1 or even higher. At 12–15 h post-infection, viral DNA replication commences and late-strand RNA begins to accumulate rapidly and almost exponentially, while early-strand RNA and proteins accumulate much more slowly. In absolute terms, the amount of early-strand RNA in the cell is similar at 12 h and 24 h post infection. Thus, there is a dramatic change in the relative abundances of early-strand and late-strand RNAs; by 24 h post-infection, the early to late RNA ratio is as low as 1 to 50 [1,18,19,20]. This early–late “switch” depends absolutely on viral DNA replication. If replication is inhibited, early mRNAs continue to accumulate but late mRNAs fail to do so [11,12,19,20,21,22]. While it was thought in the field for a number of years that the early–late switch is the result of T antigen repression of the early promoter, coupled with a transactivation of the late promoter, this now seems to be incorrect. Rather, the switch appears to result from changes in transcription elongation and/or RNA stability [13,19,23,24,25]. Late RNA accumulation is regulated post-transcriptionally by what appears to be a novel RNA titration event (late gene expression from a non-replicating viral genome can be activated in *trans* by sufficient levels of late transcription from a replicating genome in the same cell) [13], while early RNA levels are regulated at least in part by antisense RNA and RNA editing (Figure 3) [13], as well as by virus-encoded miRNA [25] (see Figure 1). 

Even before the onset of viral DNA replication, however, the late-strand is actually being transcribed, but with little stable RNA accumulation. This latter phenomenon is associated with several important genomic features. At early times after infection, polyadenylation of late-strand transcripts is efficient. This generates RNAs that can be alternatively spliced to generate mRNAs for the virion structural proteins VP1 and VP3. VP2 mRNA from these transcripts is unspliced. Importantly, however, by a mechanism that remains unclear, these RNAs appear to be inefficiently exported from the nucleus to the cytoplasm and are degraded in the nucleus [26]. 

## 3. Late-Strand RNAs

How is late-strand gene expression enhanced at late times in infection? While at early times late-strand polyadenylation is efficient, this changes dramatically at late times. After DNA replication initiation, late-strand polyadenylation becomes inefficient, allowing RNA polymerase II to continue around and around the circular genome, generating giant multigenomic transcripts. Thus, the MPyV life cycle can be viewed as an interesting model of regulation of alternative polyadenylation, a phenomenon that has been studied in a variety of other systems [27,28]. Most late-strand primary transcripts are heterogeneous in size, and range from about 2.5 Kb to over 60 Kb in length [29,30,31,32,33,34]. Most are not polyadenylated [34]. Late-strand pre-mRNA molecules are processed into mature mRNAs using a highly unusual pathway that involves ordered splice site selection from precursors containing tandemly repeated introns and exons [35]. The great majority of late RNA sequences never leave the nucleus as they are removed during mRNA processing, and are subsequently degraded [30,36]. Some of these giant transcripts may also serve as precursors for the processing of viral miRNAs, one of which also downregulates the pro-apoptotic factor Smad2 [25,37]. 

**Figure 4:**
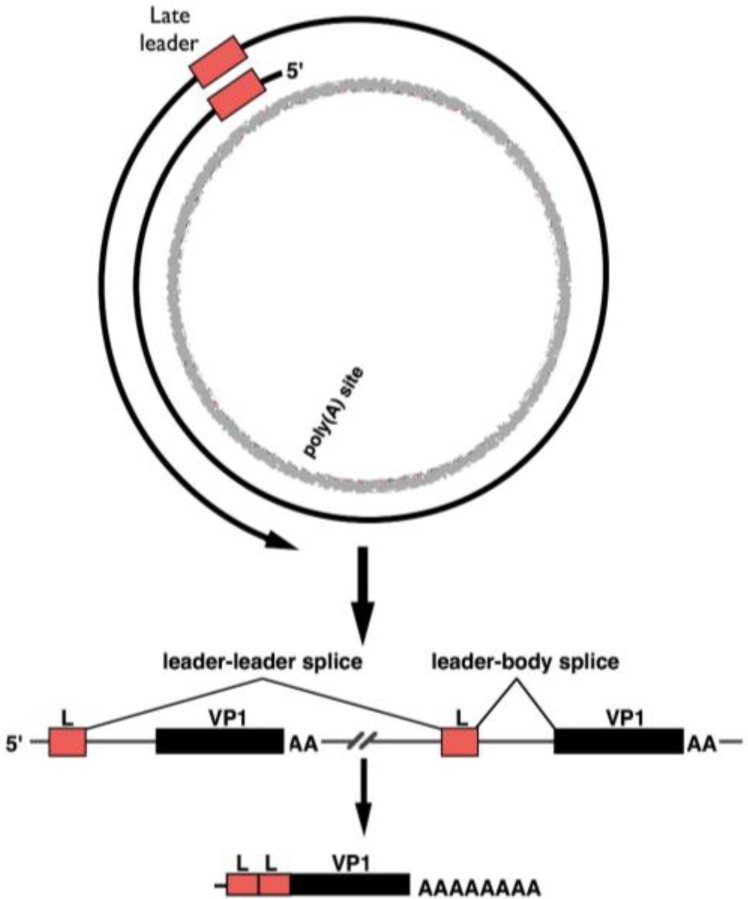
Late pre-mRNA splicing. Giant transcripts serve as precursors to late mRNAs. Processing of VP1 mRNA is shown. In multigenomic transcripts, leader (L) exons splice to one another, removing genome-length introns. Then, a leader-body splice can occur, coincident with polyadenylation. This results in mRNAs with tandem non-coding late leader exons at their 5′-ends.

The MPyV late region encodes 57-nucleotide non-coding exon near at the 5′-end of the transcription unit. At their 5′-ends, late messages contain multiple tandem repeats of this late leader sequence, which appears only once in the viral genome. Pre-mRNA molecules are processed by a pathway that includes the splicing of late leader exons to each other (Figure 4). Each class of late viral message (encoding virion structural proteins VP1, VP2 or VP3) consists of molecules with between 1 and 12 tandem leader units at the 5′-end [38], with most containing more than one. VP2 mRNA is the least abundant late message (about 5%) and contains no leader-to-body splice. Even in the absence of leader-to-body splicing, this message is nevertheless exported to the cytoplasm, although inefficiently [39]. Late-strand pre-mRNA processing is highly unusual, because it involves alternative selection between identical splice sites. Thus, in long pre-mRNAs, only the terminal coding body 3′-splice site is chosen, even though an upstream one exists in the precursors [35,38]. 

**Figure 5:**
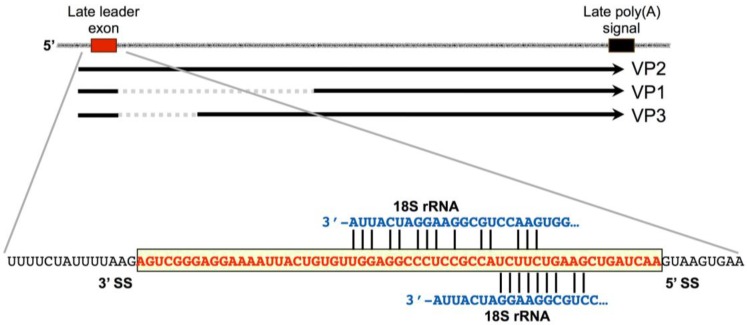
The late leader exon has regions of complementarity to 18S rRNA. While the biological consequence of this still remains unclear, there is striking complementarity to ribosomal RNA at two positions within the leader. We speculate that in late mRNAs containing tandem leaders in their 5′-untranslated regions (UTRs), these elements may serve to enhance the translation of late proteins.

While the splicing process is connected to mRNA accumulation, we hypothesize that tandem leaders may serve the additional purpose of facilitating translation initiation owing to the fact that leaders contain two regions with significant complementarity to the 3′-end of mouse 18S rRNA (Figure 5). Such regions could be coincidental and there may exist numerous other regions in cellular or viral RNAs. However, as most late MPyV mRNAs contain multiple tandem leaders in their 5′-untranslated regions (UTRs), this feature of multiple complementary sequences, preceding late AUG codons, could serve as a powerful way to recruit ribosomes and enhance the expression of virion structural proteins late in infection.

## 4. Activation of Late RNA Accumulation

Late-strand gene expression may not be regulated primarily at the level of transcription initiation. Non-replicating genomes express only very low levels of late-strand transcripts. However, late genes from these non-replicating genomes are turned on if a replicating polyoma genome is introduced into the same cell [13]. Further, the presence of wild type genomes in mouse cells can lead to the activation of late genes in *trans* from a non-replicating genome in the same cells [13]. 

## 5. The Role of dsRNA Formation and A-To-I Editing in MPyV Gene Regulation

Due to the readthrough of early and late transcripts at late times, as well as the genomic overlap of the early and late polyadenylation signals, there is the possibility that if complementary sequences accumulate near one another in the nucleus, they might anneal to form double-stranded RNAs (dsRNAs). Also, since viruses that do not allow early-strand and late-strand overlap do not undergo productive infection and cannot enter a normal late phase [16], it is likely that dsRNA plays an important role. Nuclear dsRNAs can be promiscuously edited by dsRNA-specific adenosine deaminase (ADAR) enzymes, which deaminate adenosines to inosines [40]. Consistent with sense–antisense overlap of the MPyV early and late transcripts, viral RNAs also exhibit extensive and promiscuous editing [16,20,41,42]. During productive infection, there is a time-dependent increase in editing, with especially efficient editing observed around the overlapping polyadenylation sites [20]. No significant editing was detected before DNA replication or in the presence of a replication inhibitor [16,20]. Editing in the polyadenylation region has led to speculation that this editing serves as a trigger for the early–late switch. While editing is readily and abundantly observed, however, at this time we cannot conclude whether it is a cause or a consequence of viral gene regulation. The possibility exists that editing is a consequence of dsRNA formation while duplex RNA formation may in fact be the primary event that drives the early–late switch. This is because mouse cells lacking ADAR activity have been reported to maintain the ability to support productive MPyV infection [43].

## 6. Early-Strand RNAs

How is early-strand gene expression lowered at late times in infection? There appear to be multiple mechanisms for this. While the switch from early to late phase of infection has been reported by others to be regulated primarily at the level of transcription [44], this has been challenged by results which are, in fact, consistent with a change in the processing of late-strand transcripts [14,19]. Inefficient late-strand polyadenylation and transcription termination appear to regulate early-strand gene expression in an indirect manner. The long, multigenomic late-strand transcripts in the nucleus can form RNA–RNA duplexes with early-strand transcripts which are efficient substrates for RNA editing by ADARs. This leads to the deamination of up to 50% of the adenosines on each strand to inosines (which act biochemically and genetically like guanosines). These promiscuously edited RNAs are retained in the nucleus by a quality control system involving binding to the p54^nrb^/NONO protein [45] and localization to nuclear bodies called paraspeckles [46,47], preventing them from being exported to the cytoplasm and being translated into mutant proteins [45]. As late primary transcripts accumulate to high levels in the nucleus, the opportunity for sense–antisense hybrids to form may increase, leading to ever greater inhibition by editing. While a similar phenomenon may also occur on the late strand (early-strand polyadenylation site readthrough, followed by dsRNA formation and A-to-I editing), the consequences in this case are minor because the editing would occur in genome-length introns of late pre-mRNAs rather than in late coding regions. In this manner, a cellular quality control system that prevents the nucleocytoplasmic export of dsRNAs and promiscuously edited RNAs plays an important role in reducing the availability of MPyV early-strand mRNAs for translation at late times in infection when the early gene products are no longer needed. 

**Figure 6:**
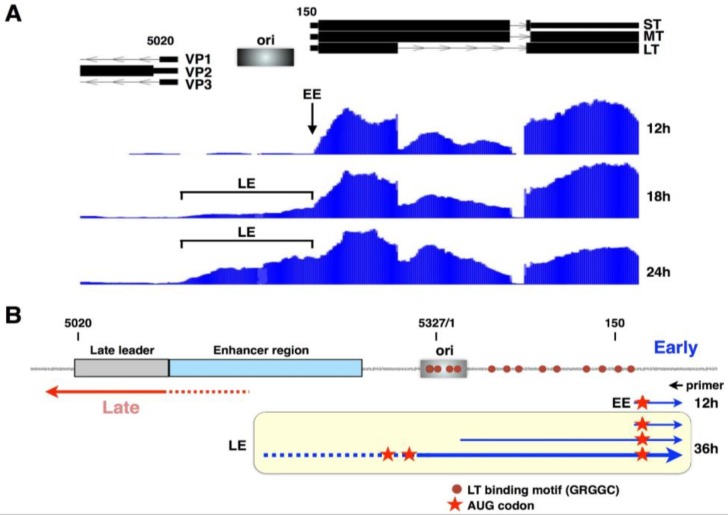
Early-strand transcription start sites shift after the onset of viral DNA replication. (**A**) An expanded view of the intergenic region is shown, along with genome browser tracks showing the alignment of early-strand RNAs at several times after infection, as reported by us recently [20]. These data were confirmed using the 5′-rapid amplification of cDNA ends (RACE) analysis [20]. Note the dramatic shift from 5′-ends mapping to a specific site at early times (EE) to many upstream sites at later times (LE); (**B**) The intergenic region is depicted, along with a general cartoon of early-strand RNAs at early times (EE) and early-strand RNAs at late times (LE). Positions of large T antigen binding are shown, along with the palindromic core replication origin, the enhancer region and the late transcription start site region. Red stars denote AUG codons that could direct translation initiation. Those in LE but not EE transcripts are frameshifted relative to the early coding region.

Yet another way in which early expression changes after the onset of viral DNA replication is at the level of transcription initiation. Through a mechanism that still remains unclear, as infection proceeds, the 5′ transcriptional start sites from the early promoter shift progressively further and further upstream from the canonical start site downstream of the early TATA element, which is used almost exclusively in the absence of DNA replication [20,22,48] (Figure 6). Thus, at late times, many early-strand mRNAs have 5′-UTRs that are hundreds of nucleotides longer than mRNAs at early times. This shift is dependent on DNA replication but not directly on the presence of large T antigen, because large T antigen is expressed in the presence of replication inhibitors, yet in this case the shift does not occur [20]. What are the consequences of shifting early-strand 5′-ends? They may alter RNA stability and therefore lead to reduced levels of mRNA. We hypothesize, however, that they serve as yet another mechanism to limit early gene expression at late times, by leading to inefficient ribosome scanning and translation initiation. We note that many of the early-strand mRNAs at late times contain AUG codons that are frameshifted relative to the normal AUG codon and therefore would be poor messages for T antigen expression. This replication-dependent switch has also been observed for several other viruses, suggesting a more common mechanism by which small DNA viruses might limit early gene expression late in infection. Altered early-strand start sites at late times have been reported both for simian virus 40 (SV40) [49] and for the John Cunningham (JC) virus [50]. 

## 7. Conclusions

In conclusion, the mouse polyoma virus employs a variety of mechanisms to regulate the synthesis, processing, stability and translation of its RNAs in order to optimize the timing and efficiency of its life cycle. Taken together, the various modes of regulation adopted have given MPyV a powerful set of strategies to ensure efficient progression through its lytic life cycle. Some of these (such as shifting transcription start sites and promoter regulation) are shared by other viruses and systems, while others (such as the role of leader-to-leader splicing, polyadenylation site overlap and nuclear retention of dsRNAs and edited RNAs) are interesting and perhaps peculiar to this virus. Interestingly, while SV40 does not normally downregulate its early gene products using antisense RNA, it has been reported that in SV40-transformed human mesothelial cells, an integrated viral genome promotes polyadenylation site readthrough from the early region, thus generating antisense RNA that downregulates late gene expression [51]. Further, a recent study on the Merkel cell polyomavirus (MCPyV) life cycle presented transcriptomic data consistent with multigenomic transcripts, similar to those we see in MPyV infection [52].
